# Daily step recommendation adherence and peak bone mineral density among female nurses: A cross-sectional study

**DOI:** 10.1097/MD.0000000000047544

**Published:** 2026-02-06

**Authors:** Guoqiong Xu, Shuang Feng, Youju Wu, Xiang Ai, Yong Zhang

**Affiliations:** a Health Medicine Center, The Second Hospital Affiliated to Chongqing Medical University, Chongqing, China; b School of Public Health, Chongqing Medical University, Chongqing, China.

**Keywords:** bone density, daily step recommendations, female, nurses, physical activity

## Abstract

This study aims to investigate the effects of popular 6000/10,000 daily step recommendations of physical activity on bone mass in young female nurses. A sample of 69 female nurses aged 22 to 35 from a teaching hospital in Chongqing, China, were invited to participate in the study. Daily walking steps were recorded by a smartphone-based application for 30 consecutive days. The participants were divided into 3 groups based on their adherence to daily step recommendations: low (average daily steps < 6000), moderate (6000 ≤ average daily steps ≤ 10,000 steps), and high (average daily steps > 10,000 steps) steps. Bone mineral density (BMD) at multiple sites (spine, hip, and heel) was measured with dual-emission X-ray scanner. Analysis of variance was used to compare BMDs in different level of daily steps, multivariable regression analysis was used to adjust the confounders. Nurses with adherence to higher step recommendation had higher means of BMDs at all bone sites, but only BMDs at spine (L1–L4, all *P* < .05) were significant. Multivariate regression analysis showed the nurses with moderate to high steps had significantly higher BMDs at the spine (*P* ≤ .001 for L1–L4) and hip (Ward triangle *P* = .047, trochanteric region *P* = .025, and intertrochanteric region *P* = .019) than those with low steps after controlling for potential confounders. However, there was no significant relationship between daily step recommendation adherence and BMD at the femoral neck (*P* = .092) or the heel (calcaneus *P* = .367). Adhere to the recommendation of 6000/10,000 steps per day without considering types of physical activity increases the bone mass in bones of spine and hip in young females.The 6000/10,000 steps daily can be recommended as a brief goal of physical activity for the prevention of osteoporosis.Cohort studies with big sample size, and diverse population are warranted to further confirm the effect of step goals on bone health.

## 1. Introduction

Osteoporosis is a prevalent bone disease affecting millions worldwide, particularly women.^[1]^ Osteoporosis is characterized by low bone mineral density (BMD) and a deterioration of bone tissue, leading to a higher risk of fractures, especially at the site of spine and hip.^[2]^ The risk of osteoporosis increases with age. However, it can also be affected by lifestyle factors such as physical activity, which determines the peak of bone mass and the speed of bone loss.^[3]^

The World Health Organization 2020 guidelines for physical activity and sedentary behavior recommend that healthy adults take at least 150 to 300 minutes of moderate-intensity aerobic activity (or equivalent) per week for substantial health benefits.^[4]^ This recommendation was also adopted by the US and Chinese physical activity guidelines.^[5,6]^ However, this physical activity recommendation regarding frequency, intensity, and duration are difficult to measure in researches and practices. Alternatively, an accelerometer or pedometer alone or integrated into other body-worn instruments can detect body motion without attention and provide an estimation of daily physical activity as a step count. Therefore, accelerometer or pedometer-based step recommendation is simple, easy to remember, and valuable for motivating physical activity by goal setting. Because devices like pedometer are not sensitive to nonambulatory activities such as cycling, swimming, and weight lifting, step-based physical activity recommendations have not fully been validated for all populations. Nonetheless, steps recommendations are still proposed informally or formally for the public and individuals. “10,000 steps per day” was first proposed as the slogan of pedometer manufacturers in the 1960s in Japan and has become very popular. Nowadays, many campaigns, programs around 10,000 steps per day have been initiated in various settings around the world for health promotion. Some studies have indicated that 10,000 steps are similar to World Health Organization guidelines if walking is the primary activity mode.^[7]^ The growing evidence also implied that 10,000 steps per day were associated with better health conditions, for instance, less body fat,^[8,9]^ low blood pressure,^[10]^ and a healthier blood lipid profile.^[11]^ However, the 10,000-step goal daily is somewhat difficult and only a 10% of adults can achieve.^[12]^ Alternatively, 6000-step daily was recommended for more sedentary persons. The current evidence also supports 6000 steps per day as a goal physical activity for health.^[13,14]^ Based on these evidence, in Dietary Recommendation for Chinese, 6000 steps per day have been formally recommended for the public to promote daily physical activity and health.^[6]^

Although many studies have evaluated various health benefits of recommended step goals of physical activity, bone health was sparely investigated. Based on our best knowledge, until now, only 1 study has been found on the relationship between step counts and bone health, in which, only calcaneus, tibia and radius were measured in middle aged Finns.^[15]^ Therefore, the effects of the popular steps recommendation on BMD in adults remain uncertain, especially in different population and different of bones. Our study aims to investigate the effects of those 2 step goals (6000 and 10,000 steps daily) on multiple sites of BMDs in young female adults living free.

## 2. Methods

This is a cross-sectional study in a real world. Steps of 30 routine days in free living were used to present a subject’s long-term adherence to the step goal (6000 or 10,000 steps daily), and then multiple-site BMDs were compared among groups with different step goal adherence.

### 2.1. Participants

Female nurses in the health management center were invited to participate in this study. The inclusion criteria were as below: age ≤ 35, not menopause, not in a condition of being pregnant or lactation, without any disease, symptoms, or medical treatment during the investigation. This study was approved by the ethics committee of the second affiliated hospital of Chongqing Medical University (no. 2022-839). Oral informed consent from all nurses was obtained before the investigation. Nurses’ general information was collected by web-based questionnaire.

### 2.2. Routine daily step measurement

A popular WeChat messaging service (Tencent Corp., China) had an add-on named WeRun, which can measure daily accumulated steps with the support of a smartphone-integrated accelerometer. Nurses were instructed to invoke (if not) WeRun, engage in their normal daily routine activities without setting any step goals, carry their phones as often as possible during work and off-work time, and share their steps on the researcher’s WeRun step ranking list through the internet. Thirty consecutive days (October 12, 2023 to November 10, 2023) were selected for step monitoring in free living condition. Researchers checked and recorded subjects’ today’s steps the next day through their WeRun ranking list.

### 2.3. Steps category

The average daily steps in 30 days were categorized into 3 groups by 2 recommended daily steps: 6000 steps daily (formal recommendation of the Chinese Dietary Guidelines),^[6]^ 10,000 steps daily (informal recommendation but widely accepted).^[7]^

### 2.4. Bone mineral density measurement

Areal BMD was measured at multiple sites, including the lumbar spine (L1–L4, anteroposterior region), hip (femoral neck, Ward triangle, and trochanteric and intertrochanteric region on both sides), and heel (calcaneal). Lumbar spine and hip were measured by DPX Bravo DXA scanners (GE Healthcare, WI, USA), and heel BMD was measured by a portable calcaneal dual X-ray scanner (LM-LUX, China). Average BMDs of the dual femur were used in the data analysis.

### 2.5. Data analysis

Continuous variables were compared by using analysis of variance, as the least significant difference test was used to compare pairwise means; the chi-square test or Fisher exact was used to compare categorical variables. Multiple linear regression was used to analyze the associations between step level and BMD. A significant level was set at 0.05 for all comparisons. All statistical analyses were performed using SPSS 19 (IBM Corp., Armonk, NY).

## 3. Results

### 3.1. Participants’ characteristics

Among the 69 female nurses eligible in the center of health, 47 completed the 30-day step monitoring and BMD measurements were included in the data analysis. The average age of 47 nurses was 28.3 ± 3.1 (range is 22–35) years with a BMI of 19.4 ± 2.2 kg/m^2^. The mean total steps in 30 days were 229,071 ± 72,306 (ranging from 86,827 to 393,063). According to the daily steps recommendations of 6000 steps and 10,000 steps daily, participants were categorized into 3 groups: 16 (34.0%) were in low (<6000 steps daily), 22 (46.8%) were in the middle (6000–10,000 steps daily), and 9 (19.2%) were high (>10,000 steps daily) by their average daily steps. The characteristics of participants were reported by step level in Table 1. All characteristics were the same among the 3 daily step groups (all *P* > .05).

**Table 1:** Characteristics of participants by step levels.

Characteristics	Total (n = 47)	Low steps (<6000 step daily) (n = 16)	Middle steps (6000–10,000 steps daily) (n = 22)	High steps (>10,000 steps daily) (n = 9)	*P*
Age (yr)	28.3 ± 3.1	28.1 ± 2.7	28.9 ± 3.2	27.4 ± 3.2	.483
Height (cm)	162.8 ± 6.6	162.9 ± 4.5	160.9 ± 2.9	167.2 ± 12.3	.056
Weight (kg)	51.3 ± 5.2	50.9 ± 6.4	52.2 ± 5.0	50.2 ± 3.1	.824
BMI (kg/m^2^)	19.4 ± 2.2	19.2 ± 2.3	19.8 ± 1.9	19.0 ± 3.0	.603
SBP (mm Hg)	113.6 ± 12.1	113.1 ± 9.8	112.3 ± 13.1	117.3 ± 5	.578
DBP (mm Hg)	70.4 ± 8.1	70.3 ± 8.7	70.7 ± 7.7	69.7 ± 8.8	.947
Year of nursing	6.6 ± 3.2	6.7 ± 2.7	6.9 ± 3.5	6.0 ± 3.8	.792
Marital status					.541
0 (unmarried)	21 (44.7)	8 (50.0)	8 (36.4)	5 (55.6)	
1 (married)	26 (55.3)	8 (50.0)	14 (63.6)	4 (44.4)	
Child number					.208
0	25 (53.2)	11 (68.8)	8 (36.4)	6 (66.7)	
1	20 (42.6)	5 (31.3)	12 (54.5)	3 (33.3)	
2	2 (4.3)	0 (0)	2 (9.1)	0 (0)	

BMI = body mass index, DBP = diastolic blood pressure, SBP = systolic blood pressure.

*P* < .05 means at least 1 group is different with the other 2 groups.

### 3.2. The BMDs at multiple sites

All participants had normal BMDs in the lumbar spine (L1–L4), dual femur (femoral neck, Ward triangle, and trochanteric and intertrochanteric regions), and calcaneus, no.1 was diagnosed as having low BMD based on the T- or Z-values of BMDs in all sites measured (data not shown).

The average BMD value at different sites was summarized in Table 2 by daily step groups. Results showed that high-level steps in daily life had higher BMDs in all measured sites. However, only the average BMDs at sites of spin (L1–L4) reached statistical significance (*P* < .05), whileas means of BMD at the hip and the heel are insignificant among 3 groups. For pairwise comparisons among 3 groups, only femoral neck and calcaneus showed no differences between any 2 groups (all *P* > .05).

**Table 2:** Bone mineral density in multiple sites by daily steps (g/cm^2^).

	Total (n = 47)	Low steps (<6000 steps daily) (n = 16)	Middle steps (6000–10,000 steps daily) (n = 22)	High steps (>10,000 steps daily) (n = 9)	*P*
Lumbar spine (anteroposterior)					
L1	1.07 ± 0.10	1.04 ± 0.09^a^	1.10 ± 0.10^a^	1.13 ± 0.12^b^	<.001
L2	1.15 ± 0.14	1.08 ± 0.08^a^	1.17 ± 0.15^b^	1.21 ± 0.16^c^	<.001
L3	1.18 ± 0.12	1.12 ± 0.10^a^	1.20 ± 0.11^b^	1.27 ± 0.13^c^	<.001
L4	1.15 ± 0.12	1.08 ± 0.10^a^	1.17 ± 0.12^b^	1.19 ± 0.09^b^	.002
Hip (dual femur)					
Femoral neck	0.95 ± 0.12	0.91 ± 0.12	0.96 ± 0.13	0.99 ± 0.09	.218
Ward triangle	0.86 ± 0.13	0.81 ± 0.12^a^	0.88 ± 0.13^a,b^	0.92 ± 0.13^b^	.090
Trochanteric region	0.74 ± 0.12	0.70 ± 0.10^a^	0.76 ± 0.11^a,b^	0.79 ± 0.13^b^	.089
Intertrochanteric region	1.13 ± 0.16	1.07 ± 0.15^a^	1.13 ± 0.15^a,b^	1.21 ± 0.13^b^	.074
Heel					
Calcaneus	0.91 ± 0.06	0.90 ± 0.06	0.90 ± 0.06	0.94 ± 0.05	.364

Two groups with different superscript letter indicates that means of bone mineral density are significant different between these 2 groups.

*P* < .05 means at least 1 group is different with the other 2 groups. Values with the same superscript down the column are not significantly different at *P* < .05.

### 3.3. Multivariable analysis of daily steps and BMDs

Multivariable regression analysis was used to explore the relationships between daily step level and BMD. In model 1 without adjusting of any covariates, daily step level was positively related to BMDs at spin (L1–L4), hip (Ward triangle, and trochanteric and intertrochanteric regions), while not to the femoral neck and calcaneus. When adjusted by covariates such as height, weight, and age, the regression coefficients remained significant and stable for all spine sites, Ward triangle, trochanteric and intertrochanteric regions, while relationships between daily steps on the femoral neck and calcaneus were still not found (Table 3).

**Table 3:** The linear regression coefficients between step level (from low to high) and bone mineral density.

	Model 1	Model 2	Model 3	Model 4
Lumbar spine				
L1	0.077 ± 0.017* (*P* < .001)	0.069 ± 0.017* (*P* < .001)	0.064 ± 0.017* (*P* < .001)	0.069 ± 0.017* (*P* < .001)
L2	0.102 ± 0.023* (*P* < .001)	0.096 ± 0.024* (*P* < .001)	0.091 ± 0.024* (*P* < .001)	0.091 ± 0.025* (*P* < .001)
L3	0.094 ± 0.020* (*P* < .001)	0.087 ± 0.020* (*P* < .001)	0.086 ± 0.020* (*P* < .001)	0.088 ± 0.020* (*P* < .001)
L4	0.081 ± 0.021* (*P* < .001)	0.078 ± 0.022* (*P* < .001)	0.079 ± 0.021* (*P* = .001)	0.081 ± 0.022* (*P* = .001)
Hip (dual femur)				
Femoral neck	0.045 ± 0.023 (*P* = .055)	0.048 ± 0.023* (*P* = .042)	0.040 ± 0.021 (*P* = .080)	0.039 ± 0.023 (*P* = .092)
Ward triangle	0.056 ± 0.027* (*P* = .041)	0.066 ± 0.026* (*P* = .020)	0.058 ± 0.025* (*P* = .037)	0.054 ± 0.025* (*P* = .047)
Trochanteric region	0.050 ± 0.021* (*P* = .023)	0.060 ± 0.021* (*P* = .009)	0.051 ± 0.020* (*P* = .019)	0.049 ± 0.020* (*P* = .025)
Intertrochanteric region	0.068 ± 0.028* (*P* = .020)	0.080 ± 0.028* (*P* = .008)	0.074 ± 0.027* (*P* = .014)	0.074 ± 0.028* (*P* = .019)
Heel				
Calcaneus	0.015 ± 0.012 (*P* = .223)	0.011 ± 0.012 (*P* = .387)	0.011 ± 0.012 (*P* = .384)	0.012 ± 0.013 (*P* = .367)

Model 1 = unadjusted, Model 2 = adjusted by age, education and nursing years, Model 3 = model 2 plus body mass index and blood pressure, and Model 4 = model 3 plus marital status and child number.

* *P* < .05 means there is a linear relationships between step level and bone mineral density.

## 4. Discussions

This study investigates the BMDs at multiple sites in young female adults aged 22 to 35. Previous studies have shown that about 95% peak BMDs were achieved by age 20,^[16]^ and around100% peak BMD the age of 30 in female^[17]^; Besides, a study about Chinese women suggested that peak BMD occurred at ages 20 to 24 at the trochanter and Ward triangle and 35 to 39 years at the anteroposterior lumbar spine, femoral neck, and forearm ultra-distal region.^[18]^ Therefore, the BMDs measured in this study can be treated as the peak BMDs of participants. Lower peak BMDs at a young age may be the most critical factor leading to the early onset of osteoporosis in the elderly, which is a major worldwide public health problem.

When categorizing the routine daily steps of 30 consecutive days by physical activity recommendation of 6000 and 10,000 steps per day, data showed that higher daily step recommendation adherence had higher BMD in young adult women, especially at the site lumbar spine and hip with statistical significance even after adjusting some covariates such as age, body mass index, blood pressure, marital status, and childbearing history. These results indicated that adherence to both step goals (6000 and 10,000 steps daily) for young female adults increased peak BMD than those who did not reach at least 6000 steps daily, which may help reduce the risk of osteoporosis comparing with those sedentary peers.

However, we did not identify any effects of the 6000 and 10,000-step adherence on the heel bone (calcaneus). It is also worth noting that heel BMD was not correlated to hip and spine BMDs in our studies (Table S1). However, other study showed that calcaneus BMD was moderately correlated to axial BMD in women with an average age of 55.^[19]^ The differences of steps or physical activities on different bones may be explained by the structures of different bones. For example, the percentages of trabecular bone (which is more metabolically active than cortical bone) at the calcaneus, hip, and spine, which are 95%, 40%, and 66%, respectively.^[20]^ Calcaneus with more trabecular bone may not be such sensitive to physical activity at a younger age, but this assumption needs further clarification.

Physical activity is an essential component of bone health. Studies found that greater BMDs were observed in gymnasts versus non-gymnasts,^[21]^ and in dominant versus nondominant arms of racquet sports players.^[22]^ Although accelerometer are not sensitive to nonambulatory activities such as cycling, swimming, and weight lifting, some studies have indicated that 10,000 steps are similar to World Health Organization guidelines about physic activity if walking is the primary activity mode.^[7]^ However, there is scarce evidence about steps on bone health, except the study by Tolonen et al,^[14]^ in which women (aged 31–46) but not men in the 4th tertile group of steps (>8765 steps per day) had beneficial traits at calcaneus, tibia and radius. Our study further confirmed the beneficial effect of habitual 6000 and 10,000 steps per day on the peak BMDs of multiple site of bones, including lumbar (L1–L4), femur (Ward triangle, trochanteric and intertrochanteric regions) in young females.

Regarding the physical activity recommendations, accelerometer-based step recommendations are simple, easy to remember, and therefore valuable for physical activity promoting by goal setting. Although the 10,000-step goal had more benefits than the 6000-step goal in our study, only 19.0% reached the goal of 10,000 steps in a free-living context in our study. The 10,000 steps per day may be achievable but a challenging for most people in routine activities. Similarly, in a large sample size study of the free-living person buying Withings tracker, 10.8% and 18.6% of young subjects (aged 18–30) reached the 10,000 goal and 7500 to 10,000 steps, respectively.^[12]^ Another study found that only 15% took at least 10,000 steps per day, and 22% took 7000 to 10,000 steps per day in a week among peripheral artery disease patients (average age was 65) when they were not given any step goals and blinded to the steps they had taken.^[23]^ The proportions of people meeting the 6000 or 10,000 step goal were higher in this study than others because nursing is an occupation that requires more walks in the workplace. Due to the benefits when adherence to the recommended step goals on bone mass and osteoporosis, more efforts to prompt physical activity to reach the daily 6000 or 10,000 steps goal for young female in the workplace and/or daily life are still needed for the long-term benefits on bone health by increasing peak BMDs.

### 4.1. Strengthening and limitations

There are some strengths in this study. Based on our best knowledge, this study is the first time to investigate the adherence of steps recommendation on bone mass in multiple bone sites, especially the spine and hip which are the more vulnerable of bone fracture. We measured multiple site BMDs with DXA methods, which allowed us to comprehensively understand the benefits of the popular daily step recommendations on various bones. Furthermore, taking steps for 30 consecutive days can eliminate fluctuations of daily activities and stably reflect the habitual physical activities. Unlike intervention studies, this study is an real world study; it evaluated the effects of step recommendations adherence under complicated real-life conditions, the findings can be referred as evidence for physical activity goal setting.

Some limitations also need to be noticed. First, the sample size is relatively small; nevertheless, as a preliminary study, we got significant results, the sample size at least is enough in this setting to detect the some effects of the step recommendation adherence on multiple sites of bones such as spine and hip bones. Study with big sample in future may help detect the effects on more sites of bones. Second, we only adjusted several covariates in the multivariate analysis. Other factors, such as diet, nutrition, alcohol, sleeping, were not investigated and included. However, participants in our study from the same department had high homogeneity concerning social and economic characteristics and working conditions; those unknown covariates may be evenly distributed among groups. Third, because of the homogeneity of participants, our results may not be generalized to other populations, further studies with various population are warranted. Fourth, the various brands of smartphone were used, which may introduce measurement bias. However, in the study by Yao et al,^[24]^ no matter what brands of smartphone used, the Wechat steps are still highly correlated with the measurements of ActiGraph-GT3X accelerometer(*R* = 0.733), which has be recognized as the gold standard of body movement. Therefore, the measurements by different brands of smartphone are reliable.

## 5. Conclusions

In the real world, adhering to the general recommendation of 6000 or 10,000 steps per day can significantly benefit young female adults on bone mass compared to those of more sedentary. The 6000- or 10,000-step per day can be used as a goal in physical activity promotions or interventions in workplaces and communities for the prevention of osteoporosis. However, intervention or cohort studies with big sample size, and diverse population are warranted to further confirm the effect of step goals on bone health.

Supplemental Digital Content “Table S1” is available for this article (http://links.lww.com/MD/R309).

## Author contributions

**Conceptualization:** Guoqiong Xu, Yong Zhang.

**Data curation:** Guoqiong Xu, Shuang Feng, Youju Wu, Xiang Ai.

**Formal analysis:** Yong Zhang.

**Writing – original draft:** Guoqiong Xu, Yong Zhang.

**Writing – review & editing:** Guoqiong Xu, Shuang Feng, Youju Wu, Xiang Ai, Yong Zhang.

## Supplementary Material


