# Supplementary material for: Daily step recommendation adherence and peak bone mineral density among female nurses: A cross-sectional study
Source: Medicine (Baltimore). 2026 Feb 6;105(6):e47544. doi: 10.1097/MD.0000000000047544 (PMC12885712; doi:10.1097/MD.0000000000047544)
Supplement: Supplementary file 1 [file medi-105-e47544-s001.pdf]

Supplementary materials

Table s1 the correlations of bone mineral density between different sites

	L1	L2	L3	L4	Femoral neck	Ward's triangle	Trochanteric region	Intertrochanteric region	Calcaneus
L1	1	**0.717****	**0.786****	**0.683****	**0.435****	**0.391****	**0.402****	**0.435****	0.003
L2		1	**0.764****	**0.600****	**0.360***	**0.348***	**0.341***	**0.332***	-0.024
L3			1	**0.883****	**0.447****	**0.429****	**0.414****	**0.475****	0.084
L4				1	**0.496****	**0.457****	**0.465****	**0.541****	0.042
Femoral neck					1	**0.869****	**0.755****	**0.832****	0.05
Ward's triangle						1	**0.877****	**0.828****	0.037
Trochanteric region							1	**0.873****	-0.014
Intertrochanteric region								1	-0.032
Calcaneus									1

**, $p < 0.01$; *, $p < 0.05$
